# Supplementary material for: Multivariate Analysis of Protein–Nanoparticle Binding Data Reveals a Selective Effect of Nanoparticle Material on the Formation of Soft Corona
Source: Nanomaterials (Basel). 2023 Nov 4;13(21):2901. doi: 10.3390/nano13212901 (PMC10647827; doi:10.3390/nano13212901)
Supplement: Supplementary file 1 [file nanomaterials-13-02901-s001.zip › nanomaterials-2682954-supplementary.pdf]

Multivariate analysis of protein-nanoparticle binding data reveals a selective effect of the nanoparticle material on the formation of soft corona

Susannah Emily Cornwell, Sarah Ogechukwu Okocha and Enrico Ferrari
Department of Life Sciences, University of Lincoln, Lincolnshire, Lincoln LN6 7TS, UK

Supplementary Materials

	gold nanoparticles	silica nanoparticles
Diameter ± SD	96 ± 11 nm	100 ± 30 nm
Coefficient of variation	11.9 %	10-15 %
Hydrodynamic diameter	104 nm	115 nm*
Zeta potential (pH 7.0)	-34 mV	-42 mV*
Particle surface	Bare (citrate)	Bare (SiOH)

Table S1. *Physical and chemical characterization of the nanoparticles used*. The diameter and the coefficient of variation were measured by transmission electron microscopy whereas the hydrodynamic diameter and the zeta potential were measured using a Zetasizer Nano ZS (Malvern). Unless indicated by an asterisk (*), the values were provided by the supplier.

Protein type	Nanoparticle material	K_D 95% c.i. (mg mL^{-1})	τ_{max} 95% c.i. (nm)
SA	Silica	0.028 – 0.177	4.4 – 8.1
	Gold	0.038 – 0.136	20.0 – 34.9
TF	Silica	0.258 – 0.731	10.8 – 19.9
	Gold	0.001 – 0.020	10.7 – 19.5
PT	Silica	0.140 – 0.298	15.1 – 21.4
	Gold	0.009 – 0.023	16.7 – 21.9

Table S2. *Confidence intervals (c.i.) of the estimates of K_D and τ_{max}*. The estimates have been obtained from the binding isotherms of Figure 1. The confidence intervals are referred to the estimates of Table 1.

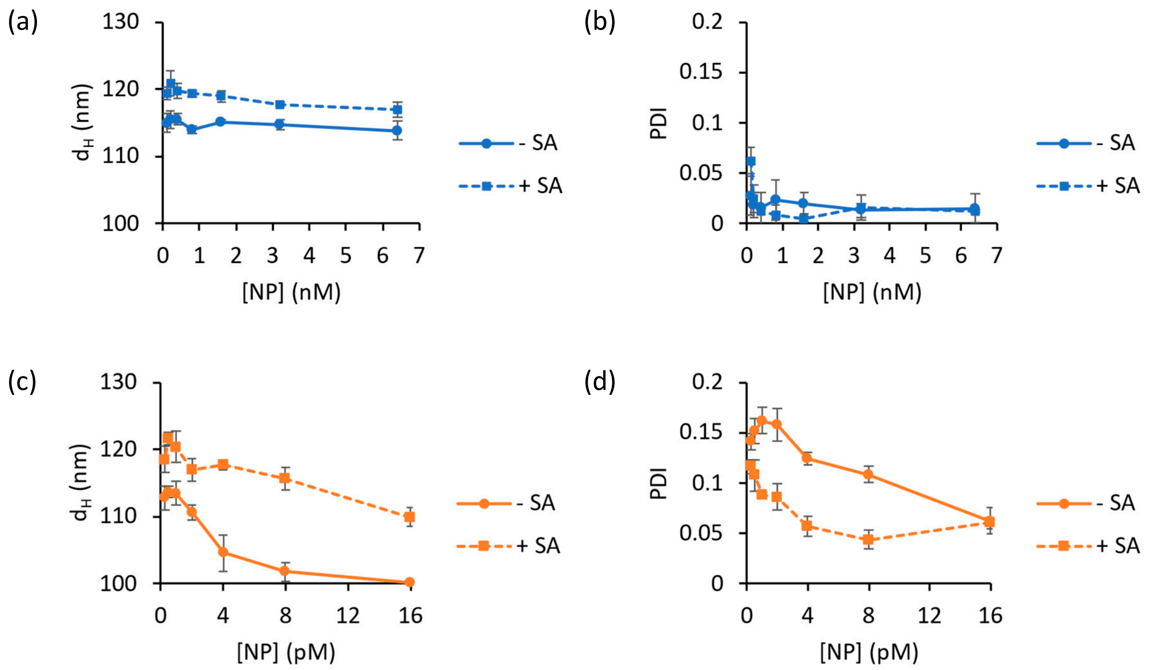

Figure S1. *Optimization of the concentration of the nanoparticles.* (a) Hydrodynamic diameter (d_H) measured in a range of silica nanoparticle concentrations in the absence (- SA) and presence (+ SA) of 0.5 mg mL^{-1} serum albumin and (b) the dimensionless polydispersity index (PDI) of the same solutions. (c) and (d) show d_H and PDI trends respectively for the gold nanoparticles. Error bars represent the standard deviation over 3 replicates.

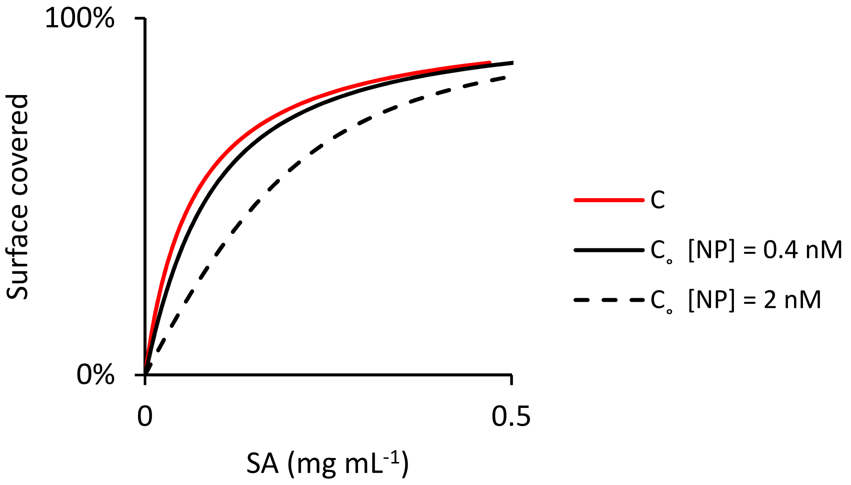

Figure S2. *Protein-nanoparticle binding curve with and without protein depletion*. The curve in red was calculated from the data in Figure 1a and taking into account protein depletion, so that the concentration of the unbound protein (C) is plotted on the horizontal axis and the vertical axis represents the percentage of coverage, which is independent of the concentration of the nanoparticles. The black solid line represents the same calculation but the total protein concentration (C_0) is plotted on the horizontal axis instead of C, and the small mismatch with the red line is due to the depletion, which is nanoparticle (NP) concentration-dependent. This mismatch would have been more substantial if the concentration of the nanoparticles was 5 times higher (2 nM, dashed line), yielding an overestimation of the dissociation constant. 2 nM is an arbitrary concentration that was used to re-calculated the curve in Figure 1a in a different scenario without nanoparticle concentration minimization.
